# Short-term prognostic analysis of patients with systemic lupus erythematosus co-infection and comparison of mNGS and conventional microbiological test results

**DOI:** 10.3389/fcimb.2023.1131258

**Published:** 2023-03-27

**Authors:** Xi Zhao, Ming-Xuan Duan, Yan-Yu Lu, Lin-Peng Bai, Xiao-Yan Zhao

**Affiliations:** Department of Cardiology, Cardiovascular Center, Henan Key Laboratory of Hereditary Cardiovascular Diseases, the First Affiliated Hospital of Zhengzhou University, Zhengzhou, Henan, China

**Keywords:** metagenomic next-generation sequencing, conventional microbiological testing, systemic lupus erythematosus, infection, short-term prognosis

## Abstract

**Objectives:**

Infection is one of the major causes of morbidity and mortality in patients with systemic lupus erythematosus (SLE), and as a new diagnostic technique, metagenomic next-generation sequencing (mNGS) is increasingly used for the pathogenetic detection of co-infected SLE patients. However, conventional microbiological testing (CMT) is still the gold standard for pathogenic diagnosis, and the specific diagnostic efficacy of mNGS versus CMT in such patients is not known. In addition, there are few studies on the short-term prognosis of co-infected SLE patients.

**Methods:**

This study retrospectively included 58 SLE patients with co-infection admitted to the First Affiliated Hospital of Zhengzhou University from October 2020 to August 2022. Patients were divided into a survivors (n=27) and a non-survivors (n=31) according to their discharge status. Baseline characteristics and etiological data were collected and statistically analyzed for all patients during their hospitalization. The sequential organ failure assessment (SOFA) score, acute physiology and chronic health evaluation (APACHE) II and systemic lupus erythematosus disease activity index (SLEDAI) were calculated for each patient to assess the predictive ability of the 3 scores on the short-term prognosis of SLE patients. The mNGS and CMT culture results were also compared to clarify the flora characteristics of patients with SLE infection.

**Results:**

More patients in the non-survivors had renal impairment, neurological manifestations, multiplasmatic cavity effusion and gastrointestinal manifestations compared to the survivors (p < 0.05). The SOFA score, APACHE II and SLEDAI were significantly higher in the non-survivors than in the survivors (p < 0.01). There were also significant differences between the two groups in several tests such as hemoglobin, platelets, albumin, total bilirubin, C-reactive protein (CRP), procalcitonin (PCT), and complement C3 (p < 0.05). In addition, the absolute values of T lymphocytes, CD4+ T cells and CD8+ T cells were smaller in the non-survivors than in the survivors (p < 0.05). The most common type of infection in this study was pulmonary infection, followed by bloodstream infection. mNGS and CMT positivity rates were not significantly different among patients in the non-survivors, but were significantly different among patients in the survivors (p=0.029). In-hospital survival of patients with SLE infection could be predicted based on the SOFA score in relation to 6. For patients with SOFA <6, we recommend earlier mNGS testing to identify the pathogen and improve patient prognosis.

**Conclusions:**

For SLE patients with co-infection, in-hospital survival can be predicted based on SOFA score. For patients with SOFA <6, advising them to complete mNGS testing as early as possible may improve the prognosis to some extent.

## 1 Introduction

Systemic lupus erythematosus (SLE) is a difficult-to-evaluate multisystem autoimmune disease most commonly seen in women of childbearing age, with a prevalence ratio of approximately 9:1 between men and women (Smith and Gordon, 2010; Lisnevskaia et al., 2014). The clinical manifestations of the disease are diverse and can involve multiple systems and sites such as the cardiovascular system, central nervous system, kidneys, lungs, and eyes. Its course is mostly insidious, slow and recurrent, or it may deteriorate suddenly and even lead to acute progression or rapid death (Esposito et al., 2014). The etiology of the disease is complex and is associated with multiple mechanisms of genetic, environmental, innate and adaptive immunity (Wahren-Herlenius and Dörner, 2013). The life expectancy of SLE patients has improved considerably in the last two decades, but the mortality rate is still high, about three times higher than that of the general population (Singh and Singh, 2020). Several studies have pointed out that infections are highly associated with morbidity, hospitalization, and mortality in SLE patients and that SLE patients have a wide range of infections, with both bacterial and viral infections being common and having typical or atypical clinical manifestations. There are fewer studies on SLE patients with co-infections.

The Sequential Organ Failure Assessment (SOFA) score is commonly used to measure the severity of organ system dysfunction and failure and is primarily used to assess the acute morbidity of critical illnesses (Lambden et al., 2019; Pölkki et al., 2022). Acute physiology and chronic health evaluation (APACHE) II was developed in 1985 for critically ill patients in all disease categories in the ICU and can assess chronic health status (Sadaka et al., 2017). Systemic lupus erythematosus disease activity index (SLEDAI) is a score recommended by the 2020 Chinese SLE guidelines and is a predictor of hospitalization for infection (Petri and Genovese, 1992). In this study, the above three scores were included to investigate whether they differed between patients in the survivors and non-survivors and to explore their predictive ability for in-hospital survival of patients with SLE infection.

The metagenomic next-generation sequencing (mNGS) assay is a novel technology that has made a landmark contribution to the etiologic diagnosis of infectious diseases. Its high sensitivity allows it to detect a broader spectrum of pathogens than conventional microbiological testing (CMT) (Chen et al., 2021). However, CMT is still the gold standard for pathogenic diagnosis, and both have advantages and disadvantages in the etiologic diagnosis of infection. mNGS and CMT have unclear diagnostic positivity rates and diagnostic efficacy in SLE patients. We will investigate the characteristics of microbiota distribution in SLE patients and compare the diagnostic efficacy of mNGS with that of CMT to determine a more appropriate diagnostic strategy for early etiologic diagnosis, accurate use of antibiotics, and improved patient prognosis.

## 2 Methods

### 2.1 Study design and population

Co-infected SLE patients admitted by the First Affiliated Hospital of Zhengzhou University from October 2020 to August 2022 were retrospectively included in this study. Our inclusion criteria were: patients with a diagnosis made by a clinician and confirmed to be clinically infected after our retrospective review of each patient’s medical records, which included clinical information, imaging examinations, and microbiological test results. The type of infection was determined by referring to the Centers for Disease Control (CDC)/National Healthcare Safety Network (NHSN) Definition of Surveillance for Specific Types of Infections (2019) and Internal Medicine Practice (15th edition) (Chen Haozhu et al., 2017; Centers for Disease Control and Prevention, 2019). Exclusion criteria: 1) not meeting the criteria for “infection” as defined in this study; 2) no mNGS test or no record; 3) age <18 years old; 4) pregnant women.

This study was approved by the Ethics Committee of the First Affiliated Hospital of Zhengzhou University (approval number: 2020-KY-429). The medical research involving human subjects in this study is in accordance with the ethical principles set forth in the Declaration of Helsinki (2013).

### 2.2 Data collection

Patients were screened according to our inclusion and exclusion criteria, and then the final inclusion was further divided into survivors and non-survivors based on discharge status. We identified and collected clinical data and examination test information from the case system during the patients’ hospitalization, including gender, age, body mass index (BMI), history of smoking, history of drinking, duration of SLE, comorbidities (hypertension, type 2 diabetes, dyslipidemia, other autoimmune diseases), clinical manifestations of SLE, medication history, maximum body temperature, SOFA score, APACHE II, SLEDAI, whether tracheal intubation, tracheotomy, continuous renal replacement therapy (CRRT) and prophylactic antibiotics, ventilator use time, intensive care unit (ICU) time, hospital stay, and days of antibiotic use. To maintain baseline consistency, the laboratory values we recorded were all within 48h of the mNGS test, which included white blood cell count, hemoglobin, platelet count, glucose, potassium, creatinine, alanine transaminase (ALT), aspartate transaminase (AST), alkaline phosphatase (ALP), albumin, globulin, total bilirubin, ΔPH (absolute value of the difference between PH and 7.40), lactate, prothrombin time (PT), activated partial thromboplastin time (APTT), D-dimer, lactate dehydrogenase (LDH), creatine kinase isoenzyme (CKMB), C-reactive protein (CRP), erythrocyte sedimentation rate (ESR), procalcitonin (PCT), interleukin-2 (IL-2), interleukin-6 (IL-6), interleukin-10 (IL-10), interferon-γ (IFN-γ), Complement C3, immunoglobulin G (IgG). In addition, the SOFA score, APACHE II, and SLEDAI in this study were all the highest values during the patient’s current hospitalisation, and they represent the severity of the condition.

We also counted immunocytological indices of SLE patients, including the percentage and absolute values of T lymphocytes, B lymphocytes and NK lymphocytes. In addition, we collected the results of mNGS tests and CMT from the enrolled patients. Most patients in this study had more than one and more than one type of CMT test, and we recorded CMT results at or after the mNGS test and combined all CMT results.

### 2.3 mNGS method and process

This study was based on the NextSeq 550Dx platform (illumina, USA) for nucleic acid detection and sequencing. Patients with SLE were enrolled, and either infection site samples or peripheral blood samples (5 mL) were collected according to defined criteria. The infection site samples we collected included pericardial effusion, bronchoalveolar lavage fluid (BALF), cerebrospinal fluid, pus, and lung tissue. About 5mL of blood is collected using an anticoagulant collection container and stored and transported at room temperature. BALF, cerebrospinal fluid, pus and lung tissue need to be cryopreserved in dry sterile tubes. After proper storage and transport, samples were inactivated in a 56°C water bath for 30 minutes prior to nucleic acid extraction to reduce sample infectivity (Kampf et al., 2020; Pastorino et al., 2020; Woldesemayat et al., 2022). DNA was extracted by TIANamp Micro DNA kit after different pretreatments for different types of samples according to the manufacturer’s instructions. DNA libraries were constructed by DNA fragmentation, end-repair, adapter ligation, and PCR amplification, followed by sequencing.

Agilent 2100 bioanalyzer (Agilent, USA) and ABI StepOnePlus Realtime PCR System were used for quality control of DNA library, including internal, negative and positive controls. The internal reference is from Arabidopsis thaliana and is provided by the sequencing manufacturer to enable tracking of the entire work process and control of process quality. And 15-20 samples containing negative controls are loaded in each macrogenomics sequencing batch and are used to detect environmental, reagent, and cross-sample contamination. Positive controls are real clinical samples proven to contain known pathogens (Lu et al., 2022). High-quality sequencing data was generated by removing low-quality and short reads (<35 bp in length), and then yield reads strictly aligned to pathogen species (SDSMRN) and reads strictly aligned to pathogen genus (SDSMRNG). The list of microorganisms obtained through the above analysis process was compared with an internal background database containing microorganisms present in more than 50% of samples in the laboratory over the past three months. Suspected background microorganisms were removed. Microorganisms with SDSMRN>50 and at least 3 times higher than the control group were considered as suspected pathogens, while the SDSMRN of suspected pathogens with SDSMRN <50 should be at least 5 times higher than the control group.

### 2.4 Study outcomes and definitions

The primary outcome in this study was in-hospital death, and the secondary outcome was the type of various infections.

CMT in this study included 1) blood culture 2) microbial staining and culture 3) sputum smear 4) serological tests for Epstein-Barr virus (EBV), cytomegalovirus (CMV) 5) CMV and EBV DNA testing 6) serum (Smith and Gordon, 2010; Esposito et al., 2014)-β-D-glucan test (G test) 7) serum galactomannan test (GM test) 8) T-SPOT tuberculosis (TB) test.

### 2.5 Statistics and analysis

The Shapiro-wilk test was used to verify that the continuous variables were normally distributed. Comparative analyses were performed using Chi-square tests for categorical variables, Fisher’s exact test (when the expected value in a cell was at least <5), t-tests for two independent samples, and one-way analysis of variance (ANOV A) for continuous variables. The SOFA score, APACHE II, and SLEDAI were evaluated for their ability to discriminate the short-term prognosis of SLE patients, and the receiver operating characteristic (ROC) curves of the three scores were compared to calculate the area under the curve (AUC) and the best cutoff value. All statistical analyses were performed using IBM SPSS Statistics 27.0 (IBM, Armonk, NY, United States) and GraphPad Prism v9.0 (GraphPad Software, La Jolla, CA, USA). *P*<0.05 indicates that the differences were statistically significant.

## 3 Results

### 3.1 Results of recruitment

**Figure 1:**
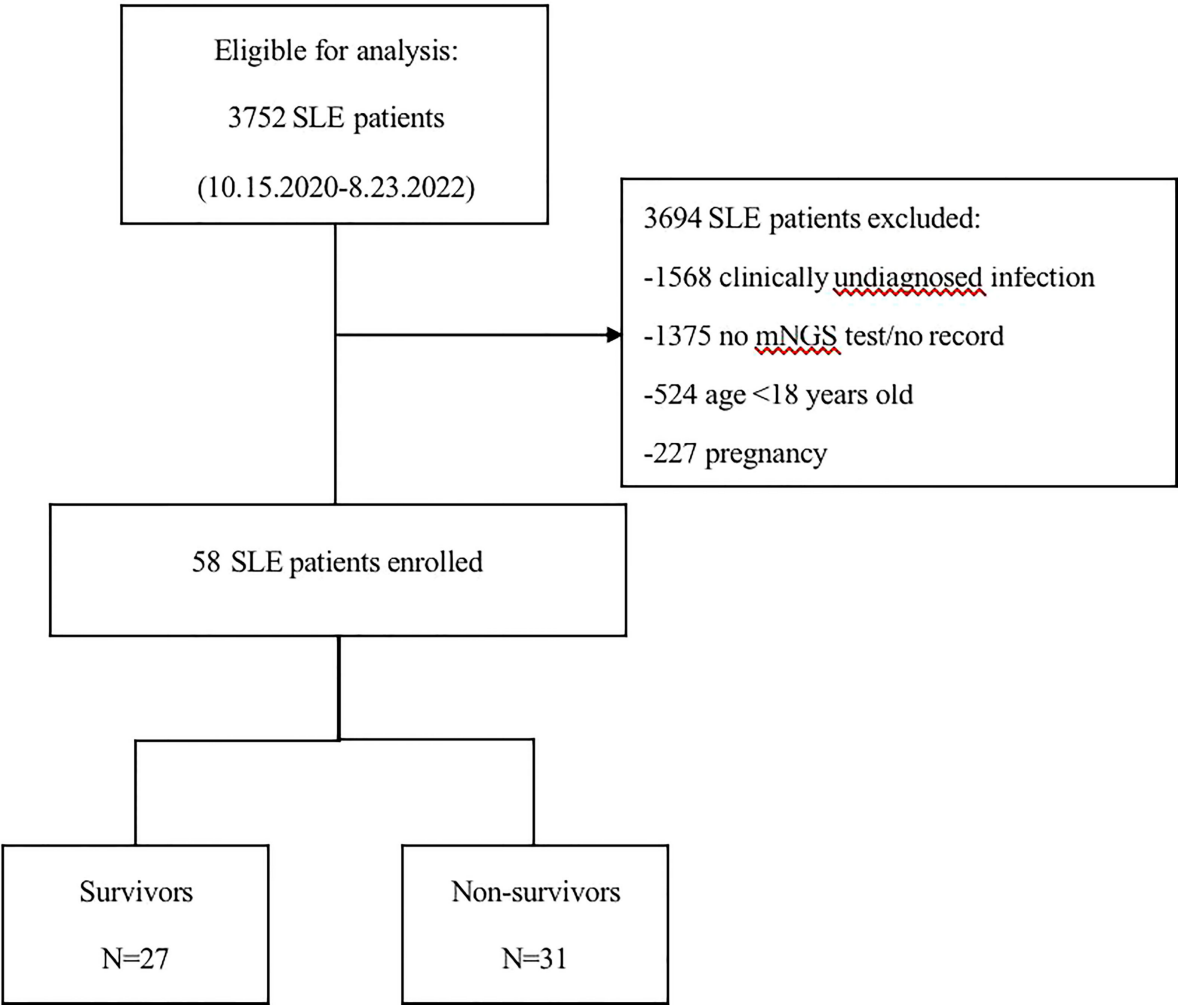
Flow chart of analyzed patients. SLE, systemic lupus erythematosus; mNGS, metagenomic next-generation sequencing.

**Figure 1** shows the screening process and clinical grouping of SLE patients. From October 15, 2020, to August 23, 2022, we reviewed 3752 SLE cases from the departments of respiratory and critical care medicine, ICU, rheumatology and infection at the First Affiliated Hospital of Zhengzhou University. A total of 3694 patients were excluded according to our inclusion/exclusion criteria, and 58 SLE patients with co-infection were included in the analysis. Based on the discharge of these patients, we divided them into a survivors (n=27) and non- survivors (n=31).

### 3.2 Clinical characteristics

**Table 1** shows the clinical characteristics of the patients recruited in this study. Of the 58 patients with SLE, 12 were male and 46 were female. The mean age was 43.6 ± 15.6 years. By analysis, we found that there were more non-survivors with hypertension (15 (48.4%) vs. 5 (18.5%), p=0.017) but no patients with combined diabetes (0 (0.0%) vs. 6 (22.2%), p=0.006) compared to the survivors group. In terms of clinical manifestations of SLE, more people in the non-survivor group had renal impairment (24 (77.4%) vs. 10 (37.0%), p=0.002), neurological manifestations (20 (64.5%) vs. 5 (18.5%), p<0.001), multiplasmatic cavity effusion (19 (61.3%) vs. 8 (29.6%), p=0.016) and gastrointestinal manifestations (27 (87.1%) vs. 12 (44.4%), p<0.001), and more people had ≥5 simultaneous systemic manifestations (22 (71.0%) vs. 4 (14.8%), p<0.001) compared to the survivor group. In terms of laboratory values, the non-survivors had lower hemoglobin (86.8 ± 20.5 vs. 98.1 ± 16.3, p=0.026), platelets (93.0 ± 64.0 vs. 181.3 ± 82.1, p<0.001), albumin (24.7 (22.1-28.6) vs. 31.9 (27.9-34.5), p<0.001), and complement C3 (0.5 ± 0.3 vs. 0.8 ± 0.4, p=0.002), while glucose (7.8 (6.0-11.0) vs. 5.2 (3.8-7.0), p<0.001), total bilirubin (12.5 (7.3-24.5) vs. 5.8 (3.9-8.9), p<0.001), ΔPH (0.1 ± 0.0 vs. 0.0 ± 0.0, p=0.009), lactate (1.7 (1.2-2.8) vs. 1.1 (0.7-1.4), p=0.006), D-dimer (3.3 (0.8-4.8) vs. 1.0 (0.3-1.9), p<0.001), LDH (581.0 (352.0-791.0) vs. 372.0 (231.0-460.0), p=0.003), CRP (68.8 (24.3-122.7) vs. 17.3 (8.2-56.0), p=0.012), PCT (1.0 (0.3-3.5) vs. 0.1 (0.1-0.4), p<0.001), IL-6 (50.7 (9.5-303.5) vs. 6.5 (3.4-11.4), p=0.010) were higher. In this study, SOFA score, APACHE II score and SLEDAI were counted separately for the survivors and non-survivors, and the results showed that all three scores were significantly different between the two groups (p<0.001). In terms of treatment course, the non-survivor group had more patients undergoing tracheal intubation (21 (67.7%) vs. 2 (7.4%), p<0.001) and CRRT (15 (48.4%) vs. 4 (14.8%), p=0.007), longer ventilator use (77.0 (24.0-191.0) vs. 0.0 (0.0-0.0), p<0.001), and longer ICU stay (8.0 (4.0-14.0) vs. 2.0 (0.0-13.0), p=0.030).

**Table 1:** Baseline characteristics of SLE patients with co-infection.

Variables	Total (n=58)	Survivors (n=27)	Non-survivors (n=31)	*P* Value
**Male**, (n%)	12 (20.7)	6 (22.2)	6 (19.4)	0.788
**Age**, (y)	43.6 ± 15.6	42.0 ± 16.1	44.9 ± 15.3	0.485
**BMI**, (Kg/M^2^)	21.9 (20.1-24.4)	21.9 (20.1-24.5)	21.7 (20.0-23.3)	0.700
**History of smoking**, (n%)	7 (12.3)	4 (14.8)	3 (10.0)	0.882
**History of drinking**, (n%)	6 (10.5)	3 (11.1)	3 (10.0)	1.000
**Duration of SLE**, (y)	2.5 (0.3-7.0)	3.0 (0.6-10.0)	1.0 (0.2-6.0)	0.223
Comorbidities, (n%)				
Hypertension	20 (34.5)	5 (18.5)	15 (48.4)	**0.017**
Type 2 diabetes	6 (10.3)	6 (22.2)	0 (0.0)	**0.006**
Dyslipidemia	36 (64.3)	14 (53.8)	22 (73.3)	0.129
Combined with other AD	9 (15.5)	4 (14.8)	5 (16.1)	1.000
SLE-related symptoms, (n%)				
Renal impairment	34 (58.6)	10 (37.0)	24 (77.4)	**0.002**
Neurological manifestations	25 (43.1)	5 (18.5)	20 (64.5)	**<0.001**
Cardiovascular manifestations	31 (53.4)	11 (40.7)	20 (64.5)	0.070
Multiplasmatic cavity effusion	27 (46.6)	8 (29.6)	19 (61.3)	**0.016**
Gastrointestinal manifestations	39 (67.2)	12 (44.4)	27 (87.1)	**<0.001**
Hematologic manifestations	51 (87.9)	21 (77.8)	30 (96.8)	0.070
Dry syndrome	3 (5.2)	1 (3.7)	2 (6.5)	1.000
Eye manifestations	1 (1.7)	0 (0.0)	1 (3.2)	1.000
Thrombosis	19 (32.8)	6 (22.2)	13 (41.9)	0.111
Types of Clinical Presentation				
≥ 5 kinds	26 (44.8)	4 (14.8)	22 (71.0)	**<0.001**
<5 kinds	32 (55.2)	23 (85.2)	9 (29.0)	
Medication History, (n%)				
Immunosuppressants	41 (71.9)	22 (81.5)	19 (63.3)	0.128
Biological agents	5 (8.6)	3 (11.1)	2 (6.5)	0.872
Laboratory examinations				
White blood cell, (10^9^/L)	7.2 (5.0-10.5)	6.5 (4.8-9.0)	7.9 (5.9-12.4)	0.107
Hemoglobin, (g/L)	92.0 ± 19.3	98.1 ± 16.3	86.8 ± 20.5	**0.026**
Platelet, (10^9^/L)	134.1 ± 84.9	181.3 ± 82.1	93.0 ± 64.0	**<0.001**
Glucose, (mmol/L)	6.6 (5.2-9.4)	5.2 (3.8-7.0)	7.8 (6.0-11.0)	**<0.001**
Potassium, (mmol/L)	3.9 ± 0.5	4.0 ± 0.7	3.8 ± 0.4	0.195
Creatinine, (µmmol/L)	78.5 (53.7-155.5)	68.0 (50.0-154.6)	83.0 (58.0-158.0)	0.350
Alanine transaminase, (U/L)	18.0 (10.5-42.5)	15.0 (7.0-41.0)	19.0 (14.0-44.0)	0.145
Aspartate transaminase, (U/L)	32.0 (16.8-53.5)	25.0 (14.0-38.0)	44.0 (18.0-61.0)	0.052
Alkaline phosphatase, (U/L)	72.0 (55.0-116.5)	69.0 (52.0-129.0)	72.0 (56.0-116.0)	0.749
Albumin, (g/L)	28.2 (23.8-32.8)	31.9 (27.9-34.5)	24.7 (22.1-28.6)	**<0.001**
Globulin, (g/L)	27.9 (22.8-31.9)	27.0 (20.4-32.0)	28.2 (24.8-31.5)	0.374
Total bilirubin, (µmmol/L)	8.2 (4.7-16.1)	5.8 (3.9-8.9)	12.5 (7.3-24.5)	**<0.001**
ΔPH	0.1 ± 0.0	0.0 ± 0.0	0.1 ± 0.0	**0.009**
Lactate, (mmol/L)	1.5 (1.0-2.0)	1.1 (0.7-1.4)	1.7 (1.2-2.8)	**0.006**
PT, (s)	11.4 (10.6-12.4)	11.3 (11.0-12.2)	11.9 (10.3-13.1)	0.743
APTT, (s)	29.7 (25.5-35.8)	29.3 (24.9-33.6)	30.3 (25.6-36.5)	0.282
D-dimer, (mg/L)	2.0 (0.6-3.5)	1.0 (0.3-1.9)	3.3 (0.8-4.8)	**<0.001**
LDH, (U/L)	425.0 (278.3-768.0)	372.0 (231.0-460.0)	581.0 (352.0-791.0)	**0.003**
CKMB, (U/L)	9.3 (4.2-19.5)	9.0 (4.4-14.1)	10.9 (3.7-24.4)	0.492
CRP, (mg/L)	39.4 (10.6-93.0)	17.3 (8.2-56.0)	68.8 (24.3-122.7)	**0.012**
ESR, (mm/h)	35.5 (16.0-69.3)	46.0 (24.5-70.5)	27.0 (11.0-74.5)	0.131
PCT, (ng/mL)	0.3 (0.1-2.4)	0.1 (0.1-0.4)	1.0 (0.3-3.5)	**<0.001**
IL-2, (pg/mL)	1.8 (1.0-2.8)	1.6 (1.0-2.2)	1.8 (0.9-3.3)	0.582
IL-6, (pg/mL)	11.4 (5.2-80.2)	6.5 (3.4-11.4)	50.7 (9.5-303.5)	**0.010**
IL-10, (pg/mL)	4.8 (2.6-8.3)	4.8 (2.1-7.9)	5.2 (2.8-9.2)	0.553
IFN-γ, (pg/mL)	1.1 (0.7-4.3)	1.3 (0.8-5.6)	1.1 (0.6-6.0)	0.866
Complement C3, (g/L)	0.7 ± 0.3	0.8 ± 0.4	0.5 ± 0.3	**0.002**
IgG, (g/L)	12.2 (9.2-15.2)	12.3 (7.7-14.6)	12.0 (9.2-15.7)	0.748
**Maximum body temperature**, (°C)	39.7 (38.8-40.2)	39.5 (37.9-40.2)	39.9 (39.0-40.2)	0.169
Severity score				
SOFA Score	6.0 (1.0-9.3)	1.0 (0.0-3.0)	9.0 (7.0-12.0)	**<0.001**
APACHE II Score	16.0 (8.0-23.0)	8.0 (5.0-13.0)	20.0 (17.0-31.0)	**<0.001**
SLEDAI, (n%)				
Mild activity	13 (22.4)	13 (48.1)	0 (0.0)	**<0.001**
Moderate activity	13 (22.4)	12 (44.4)	1 (3.2)	
Heavy activity	32 (55.2)	2 (7.4)	30 (96.8)	
Treatment measures				
Tracheal intubation, (n%)	23 (39.7)	2 (7.4)	21 (67.7)	**<0.001**
Tracheotomy, (n%)	2 (3.4)	1 (3.7)	1 (3.2)	1.000
Ventilator use time, (h)	8.8 (0.0-104.3)	0.0 (0.0-0.0)	77.0 (24.0-191.0)	**<0.001**
CRRT, (n%)	19 (32.8)	4 (14.8)	15 (48.4)	**0.007**
Prophylactic use of antibiotics, (n%)	50 (86.2)	23 (85.2)	27 (87.1)	1.000
**ICU stay**, (d)	6.6 (1.9-13.0)	2.0 (0.0-13.0)	8.0 (4.0-14.0)	**0.030**
**Hospital stay**, (d)	14.0 (8.0-24.3)	15.0 (10.0-27.0)	14.0 (8.0-20.0)	0.265
**ICU to hospital stay ratio**, (%)	48.7 (14.1-100.0)	20.7 (0.0-49.3)	90.0 (36.1-100.0)	**<0.001**
**Days of antibiotic use**, (d)	12.0 (7.8-22.0)	13.0 (8.0-27.0)	10.0 (7.0-19.0)	0.322

The data was shown as the mean ± SD, median (interquartile 25-75) or n (percentage). P values in bold meant significantly different (P<0.05). SLE, systemic lupus erythematosus; BMI, body mass index; AD, autoimmune diseases; ΔPH, absolute value of the difference between PH and 7.40; PT, prothrombin time; APTT, activated partial thromboplastin time; LDH, lactate dehydrogenase; CKMB, creatine kinase isoenzyme; CRP, C-reactive protein; ESR, erythrocyte sedimentation rate; PCT, procalcitonin; IL-2, interleukin-2; IL-6, interleukin-6; IL-10, interleukin-10; IFN-γ, interferon-γ; IgG, immunoglobulin G; SOFA, sequential organ failure assessment; APACHE, acute physiology and chronic health evaluation; SLEDAI, systemic lupus erythematosus disease activity index; CRRT, continuous renal replacement therapy; ICU, intensive care unit.

### 3.3 Immunocytological characteristics

**Table 2** demonstrates the immunocytological indices, including the percentage and absolute values of T lymphocytes, B lymphocytes and NK lymphocytes. Compared to the survivors, the non-survivors had lower absolute values of T cells (197.3 (145.2-414.8) vs. 467.9 (206.8-704.7), p=0.032), CD4+ T cells (83.4 (49.0-138.0) vs. 185.5 (86.8-360.3), p=0.005) and CD8+ T cells (111.3 (58.0-242.9) vs. 200.0 (139.8-382.8), p=0.028). However, there was no significant difference in the percentage of each lymphocyte between the two groups (p>0.05).

**Table 2:** Immunocytological indices in SLE patients with co-infection.

Variables	Total (n=58)	Survivors (n=27)	Non-survivors (n=31)	*P* Value
**T cell percentage**, (%)	74.1 (67.5-85.0)	81.6 (62.3-87.0)	71.3 (67.7-79.6)	0.251
**CD4^+^ T cell percentage**, (%)	29.2 ± 11.4	30.0 ± 10.6	28.4 ± 12.2	0.651
**CD8^+^ T cell percentage**, (%)	42.2 ± 17.1	43.3 ± 17.5	41.1 ± 17.0	0.680
**B cell percentage**, (%)	12.7 (3.7-22.4)	9.2 (1.8-16.8)	18.8 (9.5-23.8)	0.076
**NK cell percentage**, (%)	7.3 (3.5-11.0)	7.9 (4.5-11.0)	6.4 (2.8-11.6)	0.358
**T cell absolute count**, (/µL)	275.0 (149.5-616.3)	467.9 (206.8-704.7)	197.3 (145.2-414.8)	**0.032**
**CD4^+^ T cell absolute count**, (/µL)	121.9 (56.7-205.3)	185.5 (86.8-360.3)	83.4 (49.0-138.0)	**0.005**
**CD8^+^ T cell absolute count**, (/µL)	159.0 (81.0-358.5)	200.0 (139.8-382.8)	111.3 (58.0-242.9)	**0.028**
**B cell absolute count**, (/µL)	50.3 (18.2-96.2)	37.0 (16.8-125.4)	55.3 (21.7-89.0)	0.808
**NK cell absolute count**, (/µL)	24.2 (10.4-67.4)	39.0 (20.0-82.2)	21.6 (7.0-33.9)	0.060

The data was shown as the mean ± SD or median (interquartile 25-75). P values in bold meant significantly different (P<0.05). SLE, systemic lupus erythematosus; NK cell, natural killer cell.

### 3.4 Infection type distribution

**Figure 2:**
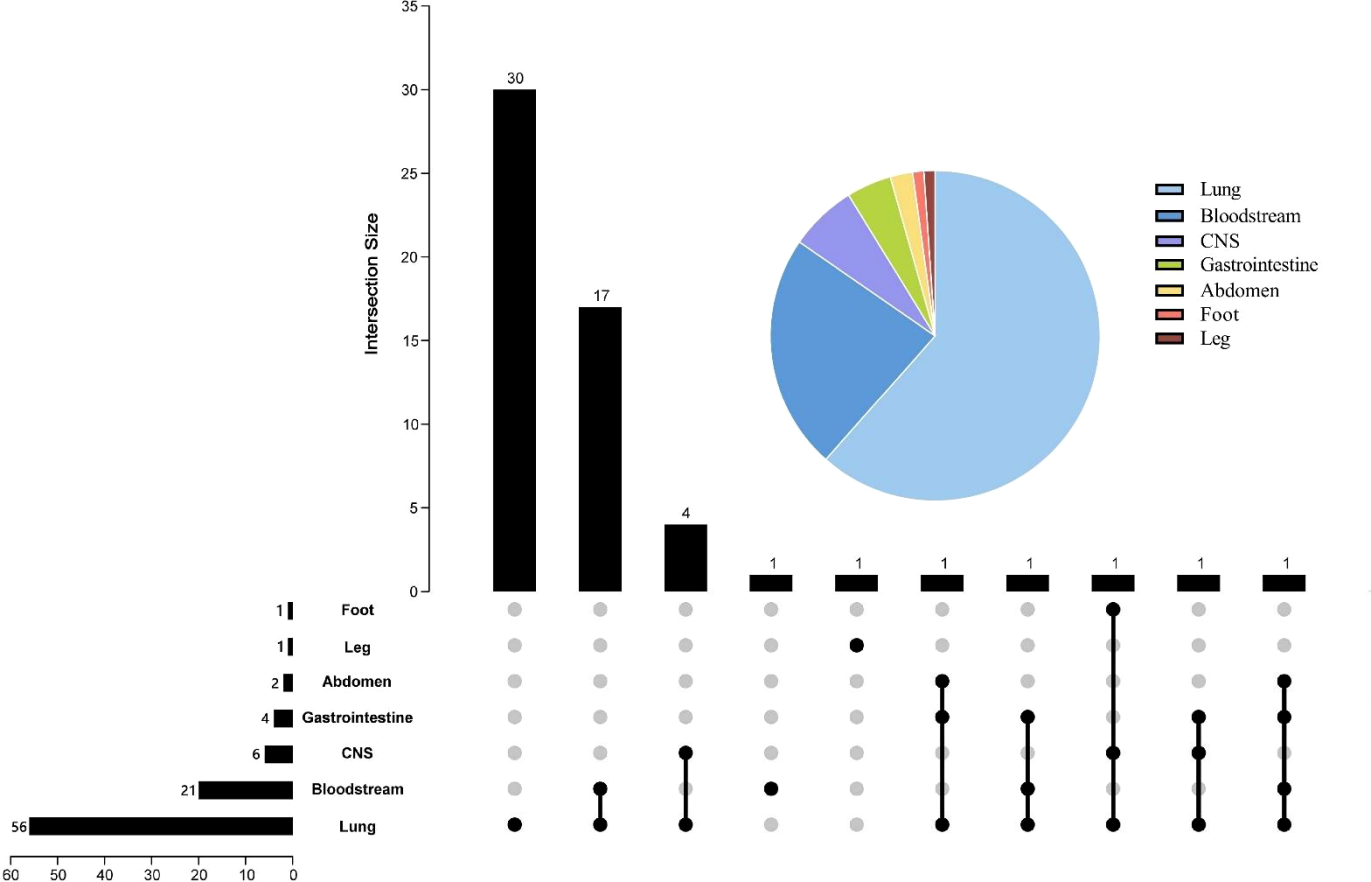
Pie and Upset charts of the distribution of infection sites. The pie chart in the upper right corner represents the percentage distribution of each infection site. In the Upset diagram, the rows represent the site of infection, and the number in front of each row indicates the total number of cases of infection at that site; the columns indicate the number of cases for each condition, and the number at the beginning of each column indicates the case of infection at the location of the “black dot”; the black dots connected by lines indicate the presence of multiple infections. CNS, central nervous system.

All 58 patients in this study were diagnosed with clinical infections, and **Figure 2** demonstrates the distribution of infection types in all patients. Of the patients, 56 were diagnosed with severe pneumonia or pulmonary infection, 21 with bloodstream infection, 6 with intracranial infection, 4 with gastrointestinal infection, 2 with abdominal infection, and finally, 1 each with leg and foot infection. The pie chart shows that pulmonary infections were the most common, with more than half of the patients having pulmonary infections. Of these, 30 patients had pulmonary infections alone and 17 patients had pulmonary infections combined with bloodstream infections. 26 patients presented with more than one lesion infection.

### 3.5 Microorganism distribution

**Figure 3:**
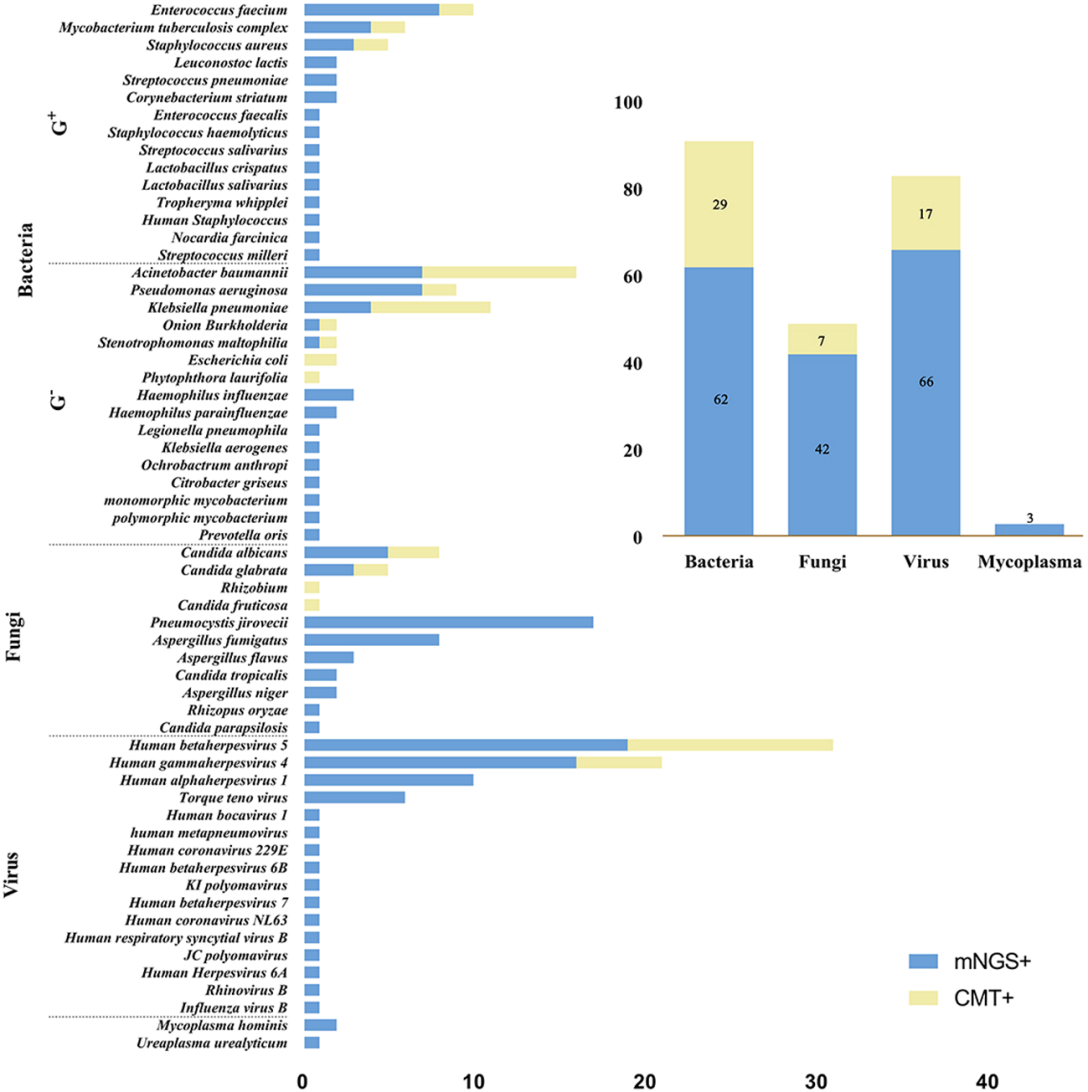
Microbial distribution bar charts. The left bar chart shows the distribution of all microorganisms detected by mNGS and CMT, and the top right bar chart shows the distribution of each type of microorganism. mNGS, metagenomic next-generation sequencing; CMT, conventional microbiological tests; G^+^, Gram-positive bacteria; G^-^, Gram- negative bacteria.

**Figure 3** shows all microorganisms detected by mNGS and CMT. Overall, mNGS was positive in 47 tests, detecting 62 bacteria, 42 fungi, 66 viruses and 3 mycoplasmas, while CMT was positive in 37 tests, detecting 29 bacteria, 7 fungi and 17 viruses. The bacteria detected by mNGS were *Enterococcus faecium* (n=8), *Acinetobacter baumannii* (n=7), *Pseudomonas aeruginosa* (n=7), *Klebsiella pneumoniae* (n=4), *Mycobacterium tuberculosis complex* (n=4), *Staphylococcus aureus* (n=3), *Haemophilus influenzae* (n=3), *Leuconostoc lactis* (n=2), *Streptococcus pneumoniae* (n=2), *Corynebacterium striatum* (n=2), *Haemophilus parainfluenzae* (n=2), *Enterococcus faecalis* (n=1), *Staphylococcus haemolyticus* (n=1), *Streptococcus salivarius* (n=1), *Lactobacillus crispatus* (n=1), *Lactobacillus salivarius* (n=1), *Tropheryma whipplei* (n=1), *Human Staphylococcus* (n=1), *Nocardia farcinica* (n=1), *Streptococcus milleri* (n=1), *Onion Burkholderia* (n=1), *Stenotrophomonas maltophilia* (n=1), *Legionella pneumophila* (n=1), *Klebsiella aerogenes* (n=1), *Ochrobactrum anthropic* (n=1), *Citrobacter griseus* (n=1), *Monomorphic mycobacterium* (n=1), *Polymorphic mycobacterium* (n=1), *Prevotella oris* (n=1); the fungi were *Pneumocystis jirovecii* (n=17), *Aspergillus fumigatus* (n=8), *Candida albicans* (n=5), *Candida glabrata* (n=3), *Aspergillus flavus* (n=3),*Candida tropicalis* (n=2), *Aspergillus niger* (n=2), *Rhizopus oryzae* (n=1), *Candida parapsilosis* (n=1); and viruses were *Human betaherpesvirus 5* (n=19), *Human gammaherpesvirus 4* (n=16), *Human alphaherpesvirus 1* (n=10), *Torque teno virus* (n=6), *Human bocavirus 1* (n=1), *Human metapneumovirus* (n=1), *Human coronavirus 229E* (n=1), *Human betaherpesvirus 6B* (n=1), *KI polyomavirus* (n=1), *Human betaherpesvirus 7* (n=1), *Human coronavirus NL63* (n=1), *Human respiratory syncytial virus B* (n=1), *JC polyomavirus* (n=1), *Human Herpesvirus 6A* (n=1), *Rhinovirus B* (n=1), *Influenza virus B* (n=1); mycoplasmas detected included *Mycoplasma hominis* (n=2) and *Ureaplasma urealyticum* (n=1). While the most frequent organisms detected by CMT were *Acinetobacter baumannii* (n=9), *Klebsiella pneumoniae* (n=7), *Pseudomonas aeruginosa* (n=2), *Escherichia coli* (n=2), *Enterococcus faecium* (n=2), *Mycobacterium tuberculosis complex* (n=2), *Staphylococcus aureus* (n=2), *Onion Burkholderia* (n=1), *Stenotrophomonas maltophilia* (n=1), *Phytophthora laurifolia* (n=1), *Candida albicans* (n=3), *Candida glabrata* (n=2), *Rhizobium* (n=1), *Candida fruticose* (n=1), *Human betaherpesvirus 5* (n=12), *Human gammaherpesvirus 4* (n=5).

**Figure 4:**
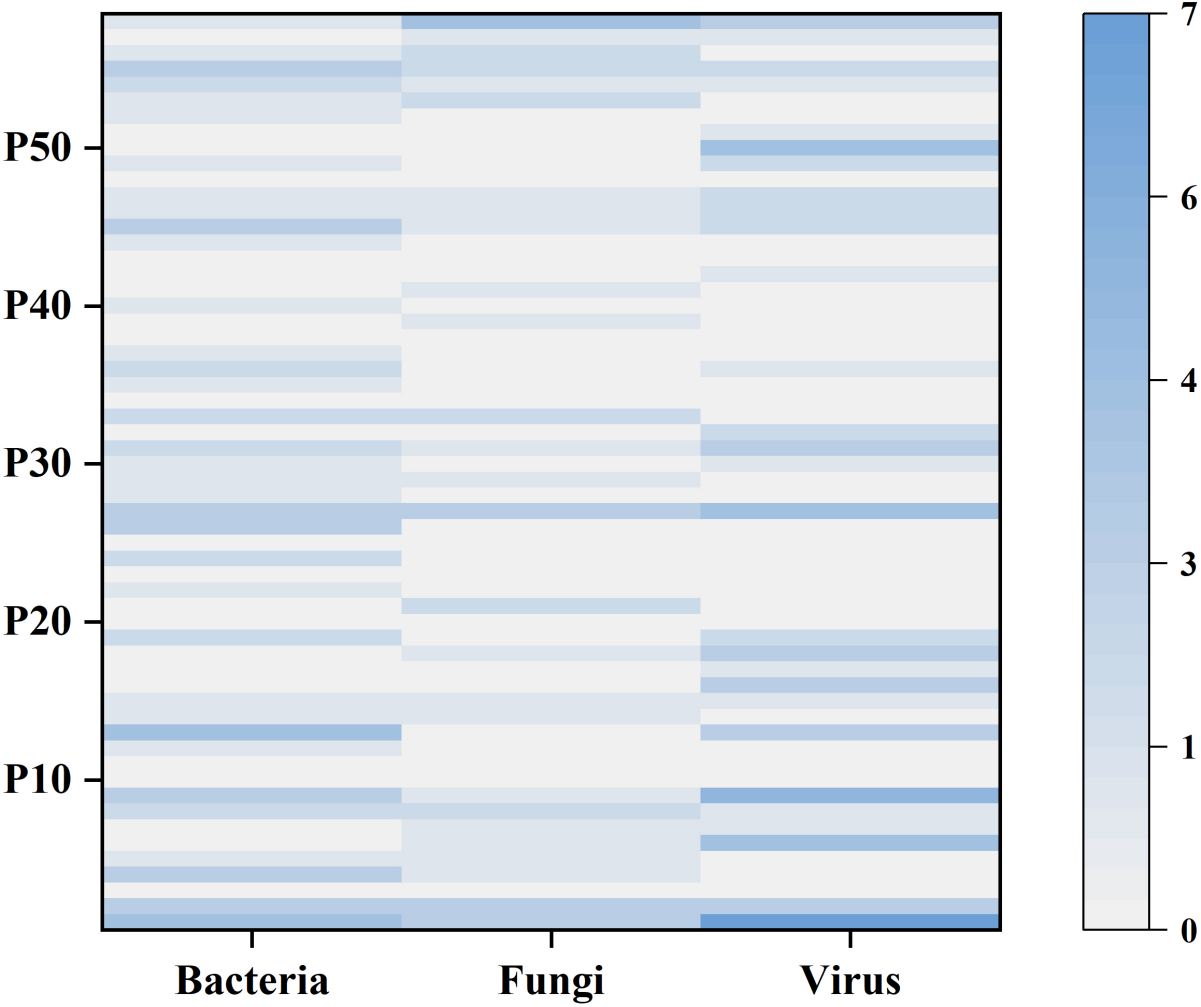
The heatmap shows the number of bacteria, fungi and viruses detected by mNGS in each sample. The left y-axis indicates the number of the 58 patients. The shade of color represents the number of microorganisms detected, with larger counts associated with darker colors.

**Figure 4** shows the number of bacteria, fungi and viruses detected by mNGS in each patient. Among all patients, 10 (17.2%) had bacterial infection only, 3 (5.2%) had fungal infection only, and 6 (10.3%) had viral infection only; 7 (12.1%) had bacterial and fungal “co-infection,” 5 (8.6%) had bacterial and viral “co-infection”, 4 (6.9%) had fungal and viral “co-infection”; and 13 (22.4%) had both bacterial, fungal and viral infections. In addition, two or more bacterial strains were detected in 16 (27.6%), two or more fungal strains in 10 (17.2%), and two or more viruses in 18 (31.0%).

### 3.6 Comparison of mNGS and CMT diagnostic performance

**Figure 5:**
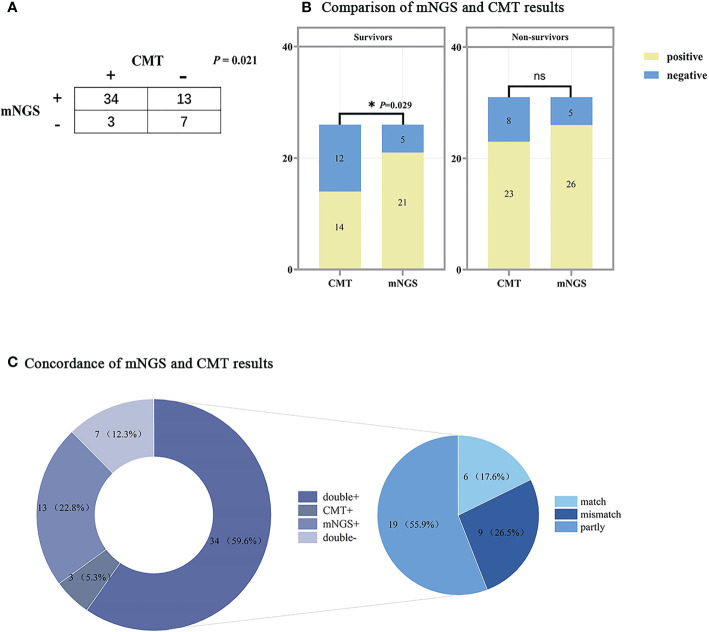
Comparison of mNGS and CMT test results. **(A)** Contingency table of the consistency of mNGS and CMT. **(B)** Bar chart of mNGS and CMT assay results in the survivors and non-survivors, the results of the two assays were significantly different in the survivors (*P*<0.05). **(C)** The pie chart shows the distribution of mNGS and CMT results for all patients. The results of the double+ group were further divided into match (6/34), partly match (at least one pathogen confirmed by the other in the test) (19/34), and mismatch (9/34). mNGS, metagenomic next-generation sequencing; CMT, conventional microbiological tests. The following are the details of the three ROC curves: SOFA score: Cut-off value=6; AUC=0.956 (95%CI: 0.905-1.000, P<0.001); Sensitivity: 93.6%; Specificity: 88.9%. SLEDAI: AUC=0.955 (95%CI: 0.893-1.000, P<0.001); Sensitivity: 96.8%; Specificity: 92.6%. APACHE II: Cut-off value=16; AUC=0.930 (95%CI: 0.861-0.998, P<0.001); Sensitivity: 90.3%; Specificity: 88.9%.

**Figure 5** shows the results of the respective tests of mNGS and CMT. Since 1 patient did not undergo any CMT, only the results of the remaining 57 patients are analyzed here. Of these, 34 (63.0%) patients were positive for both mNGS and CMT, and 7 (13.0%) patients were negative for both. Overall, the positive rate of mNGS testing was higher than that of CMT (82.5% vs 64.9%, p=0.021). The results of the two tests were analyzed separately in the survivor and non-survivor groups, and there was a significant difference between the positive rates of the two tests for patients in the survivor group (80.8% vs. 53.8%, p=0.029), while in the non-survivor group there was no significant difference between the two tests (p>0.05). Among the 34 patients who were positive for both mNGS and CMT, we further analyzed the matching of the two test results. Among them, 6 (17.6%) cases were completely matched (exact match between pathogens detected by mNGS and CMT); 19 (55.9%) were partly match (at least one microorganism overlapped between mNGS and CMT); and 9 (26.5%) were mismatch (no pathogen overlap between mNGS and CMT test results).

### 3.7 Short-term prognosis

**Figure 6:**
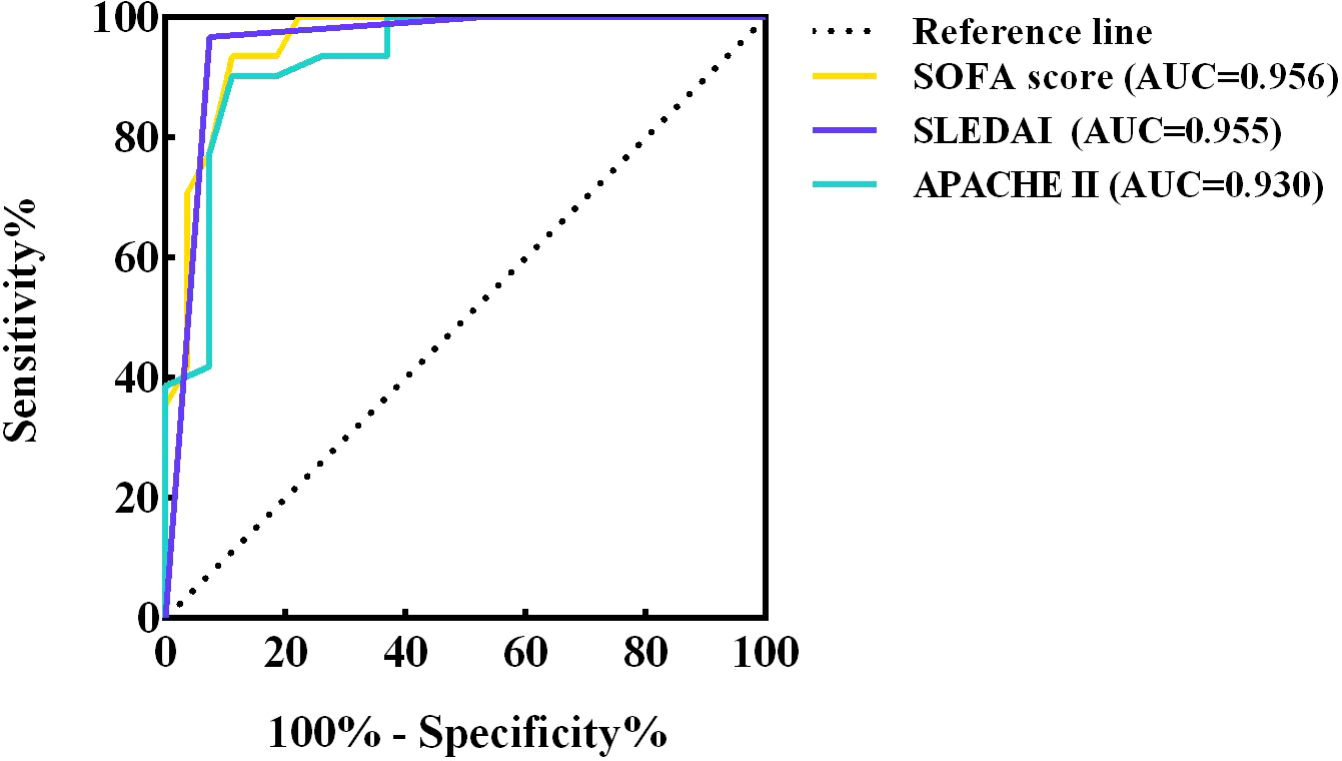
ROC curves of SOFA score, SLEDAI and APACHE II. ROC, receiver operating characteristic; AUC, area under curve ROC; CI, confidence interval; SOFA, sequential organ failure assessment; SLEDAI, systemic lupus erythematosus disease activity index; APACHE, acute physiology and chronic health evaluation.

The ROC curves of SOFA score, SLEDAI and APACHE II were plotted separately to assess their predictive ability for short-term mortality in co-infected SLE patients, yielding AUCs of 0.956, 0.955 and 0.930, respectively. The specific results are shown in **Figure 6** and the legend. The results showed that all three scores accurately predicted short-term mortality in such patients, especially the SOFA score. By calculating the maximum value of the Youden’s index, a cut-off point of 6 for the SOFA score and 16 for the APACHE II was determined.

## 4 Discussion

In recent years, several studies have shown that infection is highly associated with morbidity, hospitalization and mortality in patients with SLE. The European Lupus Project, dedicated to studying the epidemiological features as well as the clinical manifestations of SLE, found that disease activity and infection were the leading causes of death in the first 5 years of illness in patients with lupus (Cervera et al., 2009). The Hopkins Lupus and University College UK cohorts also listed infection as a cause of hospitalization and death (Petri and Genovese, 1992; Goldblatt et al., 2009). disease activity, immune system dysregulation, hormonal and immunosuppression-induced immunodeficiency, and inadmissible damage to organ systems from the disease all contribute to a much higher risk of infection in SLE patients (Rigante et al., 2014).

In this single-center retrospective study, we included 58 patients with coinfected SLE, all of whom were treated with glucocorticoids and 63.3% with cyclophosphamide or mycophenolate. Glucocorticoids have anti-inflammatory and immunosuppressive effects that interfere with leukocyte, fibroblast, and endothelial cell function and reduce the number of circulating monocytes and macrophages, leading to opportunistic infections (Staples et al., 1974; Riccardi et al., 2002). The use of immunosuppressive drugs such as cyclophosphamide may further increase the risk of infection and is still controversial (Gladman et al., 2002; Bosch et al., 2006). In addition to the ability of drugs to cause an increased risk of infection, SLE itself brings irreversible damage to the hematopoietic system, renal function, and immune system that promotes the development of infection. In addition, a significant proportion of SLE patients undergo invasive procedures, such as CRRT placement in patients with lupus nephritis, failure to maintain oxygen saturation requiring tracheal intubation, and deep venous cannulation, all of which have the potential for infection. In the present study, a greater proportion of patients in the non-survivors required tracheal intubation and CRRT, and the ventilator was used for a longer period of time, indicating, on the one hand, that patients in the non-survivors had a higher severity of disease; on the other hand, these operations may have further aggravated the infection and led to worsening of the disease instead. Our analysis of 58 patients with co-infected SLE revealed significant differences in clinical manifestations, laboratory indices, correlation scores and immunocytological indices between the survivor and non-survivor groups. Almost half of the patients in the non-survivors had a history of hypertension, and most of them had renal damage or lupus nephritis. In addition, there were more patients with combined neurological manifestations, multiple plasma membranes and gastrointestinal manifestations, which is consistent with previous studies (Wang et al., 2021). In-hospital death occurred in 71% of patients with ≥5 systems involved in SLE, suggesting that the more diverse the clinical presentation, the higher the mortality may be. In terms of test results, there were also significant differences in several indicators between the two groups, specifically concerning inflammatory indicators such as hemoglobin, platelets, albumin, total bilirubin, complement C3 and CRP and PCT. During clinical treatment, more attention should be paid to changes in these tests, which may affect the outcome of patients with SLE. manderson AP et al. concluded that the complement system hair plays an extremely important role in the pathological process of SLE (Manderson et al., 2004). Complement C3 is central in the classical and bypass pathways of the complement system, and its degradation products C3d and C4d are thought to be markers of complement activation in the inflammatory response (Ricklin et al., 2016; Deng et al., 2022). The circulatory system and endogenous immune complexes cause inflammatory cell infiltration, inflammatory mediator release, cytokine overproduction, and ultimately organ damage with increased complement C3 consumption and decreased production, increasing the risk of infection and exacerbating renal injury (Ho et al., 2001; Qi et al., 2018; Sawada et al., 2019). The predictive value of CRP and PCT for infection in patients with SLE is not yet clearly established. Some studies have suggested that SLE patients can be distinguished from SLE disease activity by determining whether they are infected based on elevated CRP, but some studies have reported conflicting results (Roy and Tan, 2001; Navarro-Zarza et al., 2010). In addition, PCT has been proposed to have a negative predictive value for bacterial infection in active SLE (Singh and Singh, 2020). Therefore, the diagnostic value of inflammatory indicators for infection in SLE patients needs to be confirmed by more studies, but in our study, inflammatory indicators such as CRP, PCT, and IL-6 were significantly elevated in the non-surviving group of patients.

The homeostasis of lymphocyte subsets is important for the immune response (Prado et al., 2013; Katsuyama et al., 2018). T cells are an important factor in the pathogenesis of SLE, and alterations in T cell signaling, cytokine production, and defects in proliferation and regulatory functions have been demonstrated in SLE patients (Crispín et al., 2010; Kaul et al., 2016). T lymphocytopenia, especially CD4+ T lymphocytopenia, is the most common hematological abnormality in SLE (Durand et al., 2000). CD4+ T lymphocytes are closely related to the body’s immunity and a decrease in CD4+ T lymphocytes often indicates an increased likelihood of opportunistic infections such as Pneumocystis carinii pneumonia (PCP) or viral infections. In this study, lymphocytes from SLE patients were counted and analyzed, and the absolute values of T lymphocytes were found to be lower in patients in the non-survivors than in those in the survivors. It is evident that SLE patients commonly experience a decrease in T lymphocytes, but to a greater extent in those with severe disease. However, there was no significant difference in the percentage of T lymphocytes between the two groups, probably because the sample size was too small for the difference to be significant. In the clinical management of SLE, we should pay attention to the above-mentioned tests with significant differences, which are inseparable from the severity of the disease, and emphasize the importance of lymphocyte typing, so that every SLE patient can have this test perfected as much as possible.

In addition to the differential tests, the SOFA, APACHE II, and SLEDAI scores were introduced in this study to investigate whether they could effectively predict the short-term prognosis of SLE patients. The AUC of the SOFA ROC curve was the largest, indicating that it is a good predictor of in-hospital survival in patients with SLE infection.

The mNGS test is used as a new technology for pathogen detection to identify new or unexpected pathogens (Hu et al., 2021). It is capable of sequencing nucleic acids from all organisms in a sample and theoretically has the ability to detect any infectious microorganism (Haslam, 2021). The traditional pathogenic tests that are often used in clinical practice today all have their own limitations. Bacterial/fungal culture assays both have long testing period and low positive rates (Guarner and Brandt, 2011). Viral assays are based on PCR with serological testing, have fixed targets, and are biased (Maartens et al., 2020). Although these traditional assays are the gold standard for pathogen detection and have sufficient evidence-based medical evidence, they are difficult to detect emerging/rare pathogens and have limited sensitivity with narrow detection targets. In contrast, mNGS is now increasingly used in clinical settings because it does not require culture and has a short reporting time, has the ability to detect 27,000 species of bacteria, fungi, viruses and parasites, and can achieve a 30% positive rate. However, due to the high positive rate and the lack of standardized testing criteria, the true and false positivity of microorganisms and the guiding significance for clinical diagnosis have not been fully affirmed. In this study, we analyzed the distribution of infection types in SLE patients and compared the diagnostic performance of mNGS with that of CMT. In terms of infection types, the most common type of infection in SLE patients was pulmonary infection, followed by bloodstream infection and intracranial infection. This differs from the study by Gladman et al, who found that the top three infection types in SLE patients were respiratory, cutaneous, and genitourinary tracts (Gladman et al., 2002). In terms of the types of microorganisms detected, *Acinetobacter baumannii*, *Klebsiella pneumoniae*, *Enterococcus faecium* were the most common bacteria, *Pneumocystis jirovecii*, *Candida albicans*, *Aspergillus fumigatus* were the most common fungi, and *Human betaherpesvirus 5* and *Human gammaherpesvirus 4* were the most common viruses. The microorganisms detected by both mNGS and CMT were dominated by bacteria and and viruses. Comparing the diagnostic results of mNGS with those of CMT, we found that the concordance rate was not high, with only 17.6% of the results overlapping completely, especially for the surviving group of patients, where there was a significant difference in the positive rate between the two tests (p=0.029). This indicates that for patients with relatively mild disease and high in-hospital survival rate of SLE infection, the bias in the test results between mNGS and CMT is relatively high; while for patients with severe disease and high mortality rate, the positive rates of the two assays are similar with no significant difference. However, it may also be that the difference is covered by severe multi-organ failure. Therefore, for patients with SLE, we can first score them, and when the SOFA score is ≥6, which means that they have a high in-hospital mortality rate, when we suspect co-infection for pathogenic testing, the efficacy of mNGS and CMT is similar, and one of them is preferred; while when their SOFA score is <6 and they are judged to have a good prognosis, mNGS with a high positive rate is recommended for pathogenic testing The mNGS test with a high positive rate is recommended for patients with a good prognosis with a SOFA score <6. Of course, for patients who are not in an emergency situation and are in a better financial position, it is recommended to improve the mNGS test and CMT to corroborate each other and better guide the treatment.

Several limitations of this study remain. Firstly, this study is a single-center retrospective study with relatively small sample sizes in both the survivor and non-survivor groups, and the results may be biased compared to studies with large sample sizes; secondly, the rigour of the conclusions could be improved as matched patients without mNGS results were not included for comparison; furthermore, due to the lack of criteria for interpreting mNGS results, the mNGS results derived in this study may lead to false positives or false negatives; finally, the majority of patients in this study had been prophylactically administered antibiotics prior to mNGS or CMT, which may have led to changes in the bacterial/fungal/viral profile and affected the pathogenic test results. More prospective and multicenter studies with large samples are needed in the future to explore the infection characteristics of SLE patients and will further investigate the interpretation criteria of mNGS results.

## 5 Conclusion

In SLE patients with co-infection, great clinical attention should be paid to their clinical manifestations, the diversity of which is closely related to the short-term prognosis of the patients. In such patients, it is important to refine lymphocyte typing, and in this study, SLE patients who experienced in-hospital death had significantly lower absolute T lymphocyte values. the SOFA score, APACHE II, and SLEDAI were all good predictors of the short-term prognosis of co-infected SLE patients. The most relevant prognosis is the SOFA score, where SOFA ≥ 6 means that the patient is much more likely to die in-hospital. mNGS and CMT positivity rates differed significantly in the survivor group, so we recommend that SLE patients with SOFA scores < 6 be tested for mNGS as early as possible. Of course, when economic conditions and practical situations allow, we recommend that the importance of mNGS testing be increased and anti-infective medication be given in conjunction with the actual situation to avoid aggravation or persistence of infection in SLE patients, thus reducing mortality and improving short-term prognosis.

## Data availability statement

The data that support the findings of the study "Short-term prognostic analysis of patients with systemic lupus erythematosus co-infection and comparison of mNGS and conventional microbiological test results" have been deposited into EMBL database with accession number PRJEB60557.

## Ethics statement

The studies involving human participants were reviewed and approved by The Ethics Committee ofthe First Affiliated Hospital of Zhengzhou University (approval number: 2022-KY-0045). Written informed consent for participation was not required for this study in accordance with the national legislation and the institutional requirements.

## Author contributions

XZ, M-XD and L-PB: software, formal analysis, data curation, writing-original draft. X-YZ and XZ: conceptualization, methodology, writing - review and editing. Y-YL: validation, formal analysis. X- YZ and XZ: term, resources, project administration, funding acquisition. All authors contributed to the article and approved the submitted version.
